# BRAF inhibitor or BRAF/MEK inhibitor treatment for patients with metastatic BRAF V600E mutated differentiated thyroid cancer

**DOI:** 10.20945/2359-4292-2025-0127

**Published:** 2025-10-23

**Authors:** Inbar Finkel, Yasmin Korzets, Assaf Moore, Tara Coreanu, Aron Popovtzer, Hagit Shoffel-Havakuk, Gideon Bachar, Jobran Mansour, Chana Weiss, Eyal Robenshtok

**Affiliations:** 1 Tel-Aviv University, Oncology Division, Tel Aviv Sourasky Medical Center, Tel Aviv, Israel; 2 Tel-Aviv University, Oncology Division, Davidoff Center, Rabin Medical Center - Beilinson Hospital, Petah Tikva, Israel; 3 Tel-Aviv University, Institute of Oncology, Davidoff Center, Rabin Medical Center - Beilinson Hospital, Petah Tikva, Israel; 4 Tel-Aviv University, Department of Otolaryngology Head and Neck Surgery, Rabin Medical Center, Petah Tikva, Israel; 5 Hebrew University of Jerusalem, Sharett Institute of Oncology, Hadassah Medical Center, Jerusalem, Israel; 6 Tel-Aviv University, Department of Otolaryngology, Head and Neck and Maxillofacial Surgery, Tel Aviv Sourasky Medical Center, Tel Aviv, Israel; 7 Tel-Aviv University, Molecular Pathology Unit, Rabin Medical Center, Petah Tikva, Israel; 8 Tel-Aviv University, Endocrinology & Metabolism Institute, Rabin Medical Center, Petah Tikva, Israel

**Keywords:** Thyroid neoplasms, proto-oncogene proteins B-raf, dabrafenib, trametinib, progression-free survival

## Abstract

**Objective:**

The aim of this study is to demonstrate the real-life efficacy of BRAF and MEK inhibitors in patients with advanced thyroid cancer.

**Subjects and methods:**

This retrospective study evaluated the clinical efficacy of either a BRAF inhibitor (dabrafenib) alone or a BRAF inhibitor (dabrafenib) in combination with a MEK inhibitor (trametinib) in the treatment of 10 patients diagnosed with metastatic BRAF-mutant RAI refractory thyroid cancer. The primary endpoint was the investigator-assessed overall response rate (ORR).

**Results:**

The median patient age was 68 years, 60% were men, and all patients were diagnosed with progressive BRAF V600E-mutant RAI-refractory papillary thyroid carcinoma (PTC). In total, 70% of the patients had been previously treated with multikinase inhibitors. One (1%) patient received a BRAF inhibitor alone and 9 (90%) patients received a combination of BRAF and MEK inhibitors. After treatment, 2 (20%) patients achieved a complete response, 5 (50%) patients achieved a partial response, 1 (10%) patient experienced stable disease, and 1 (10%) patient experienced progressive disease. Seven (70%) patients had an objective response rate (ORR) (complete or partial response). Progression-free survival (PFS) was 70%, 40%, 30%, and 30% at 6, 12, 18, and 24 months, respectively. The 12-month overall survival (OS) rate was 90%.

**Conclusion:**

Dabrafenib in combination with trametinib was well tolerated and resulted in substantial clinical benefit, with notable PFS and sustained OS, even as a second-line treatment, in patients diagnosed with metastatic, progressive BRAF V600E-mutated, RAI-refractory thyroid cancer.

## INTRODUCTION

Thyroid cancer accounts for more than 90% of all endocrine cancers (^1^), with 2%-3% of cases unresponsive to standard therapies (^2^). The most prevalent thyroid cancers include papillary thyroid carcinoma (PTC) and follicular thyroid carcinoma (FTC), accounting for approximately 94% of all thyroid cancer diagnoses (^3^).

Despite the overall favorable prognosis of differentiated thyroid cancer (DTC) (85% 10-year survival) after surgery (thyroidectomy), radioactive iodine (RAI) treatment, and thyroid hormone suppression, approximately 2%-3% of patients develop metastatic thyroid cancer that is refractory to RAI (^4^). Patients diagnosed with metastatic RAI-refractory thyroid cancer have a very poor outcome, with a 10%-29% 10-year survival rate (^5^). RAI-refractory thyroid cancer is also generally unresponsive to standard cytotoxic chemotherapy, highlighting the need for novel therapeutic approaches for this rare and aggressive cancer (^6^).

Identifying disease biomarkers through genetic and molecular biology techniques has revolutionized carcinogenesis research and led to the discovery of novel treatment targets in RAI-refractory thyroid cancer. Key pathways involved in cell proliferation, such as the mitogen-activated protein kinase (MAPK)/extracellular signal-regulated kinase (ERK) and phosphoinositide 3-kinase (PI3K)/protein kinase B (AKT) pathways, have been studied across different cancer types, and the prevalence of genetic mutations linked to these pathways has been observed in RAI-refractory thyroid cancer (^7^,^8^). Specifically, the B-type Raf kinase (BRAF) V600E mutation, which results in constitutive activation of BRAF kinase and oncogenesis, is present in approximately 29%-83% of human thyroid malignancies (^9^). Aggressive thyroid cancer and RAI resistance are associated with BRAF V600E mutations (^10^).

In recent years, the emergence of novel therapeutic options that target the mutant BRAF downstream enzyme MEK has shown promising results in metastatic melanoma (^11^) and other cancers. However, resistance to BRAF inhibitors (e.g., dabrafenib) as monotherapy in treating melanoma has been observed, and strategies involving combination therapy (BRAF inhibitors [dabrafenib] and MEK inhibitors [trametinib]) show promise (^12^). Preclinical (transgenic mouse models of BRAF V600E-mutant anaplastic thyroid cancer [ATC]) findings also indicate that the combined inhibition of BRAF and MEK improve treatment response and prevent MAPK pathway reactivation (^13^).

Previous studies have demonstrated that the combination of dabrafenib and trametinib has robust clinical activity in patients with ATC, with an overall response rate (ORR) of 69% (^14^), a 12-month duration of response (DOR) rate of 56%, a progression-free survival (PFS) rate of 43.2% and an overall survival (OS) rate of 51.7% (^15^). This led to the United States Food and Drug Administration (FDA) approval of this combination for the treatment of BRAF-mutated ATC in 2018 (^16^). In patients with DTC, a multicenter phase 2 study comparing dabrafenib monotherapy and dabrafenib plus trametinib therapy in patients with BRAF-mutated RAI-refractory DTC showed similar response rates per the Response Evaluation Criteria in Solid Tumors (RECIST) at 35% with dabrafenib and 30% with dabrafenib plus trametinib (^17^). Importantly, in 2022, the combination of dabrafenib and trametinib was approved by the FDA for all solid tumors harboring the BRAF V600E mutation, including DTC.

Currently, lenvatinib (a tyrosine kinase inhibitor of VEGFRs 1, 2, and 3) (^18^) and sorafenib (an inhibitor of VEGFRs 1, 2, and 3 and a weak inhibitor of serine/threonine kinases of BRAF) (^19^) are FDA-approved first-line therapies for metastatic RAI-resistant DTC. These medications are the backbone of treatment for patients diagnosed with metastatic thyroid cancer (^20^,^21^). However, in recent years, genetically targeted firstand second-line therapeutic options for metastatic thyroid cancer have emerged (^22^). In this context, rearrangement during transfection (RET) with tyrosine kinase inhibitors such as selpercatinib and pralsetinib (selective), as well as the neurotrophic tropomyosin receptor kinase (NTRK) fusion inhibitors larotrectinib and pralsetinib, has shown promising results (^23^).

The aim of this retrospective study was to investigate the clinical efficacy of dabrafenib alone or dabrafenib in combination with trametinib as first-, secondor third-line therapy for patients diagnosed with progressive, BRAF V600E-mutated, RAI-refractory, metastatic PTC.

## SUBJECTS AND METHODS

### Study design and ethical considerations

This retrospective study included all patients aged > 18 years who were diagnosed with metastatic BRAF V600E-mutated RAI-refractory thyroid cancer between 2008 and 2024 at the Rabin Medical Center (RMC) or Sourasky Medical Center in Israel. A total of 10 patients outside of a clinical study were identified and comprised the study group. All patients received the treatment as part of routine care. The appropriate ethics committee or Institutional Review Board (IRB) approved the study protocol. The study was conducted in accordance with the guidelines for good clinical practice (GCP) and the ethical principles described in the Declaration of Helsinki, following all applicable local regulations.

Study data were analyzed according to 3 therapeutic groups: patients treated with either first-, secondor third-line therapy.

In the first-line group, 5 patients received lenvatinib, 2 patients received sorafenib, and 3 patients received the dabrafenib plus trametinib combination. In the second-line group, 1 patient received lenvatinib, 1 patient received dabrafenib, and 5 patients received the dabrafenib plus trametinib combination. In the third-line group, 1 patient received lenvatinib and 1 patient received the dabrafenib plus trametinib combination. Patients who continued to receive secondor third-line therapy received first-line therapy with lenvatinib or sorafenib as previous therapy. The dosage for dabrafenib was 150 mg twice daily and that for trametinib was 2 mg once a day. Treatment modifications were made by head and neck oncologists experienced in systemic therapy for thyroid cancer.

### Clinical evaluations

Data on oncological assessments, active treatment, and date of death were collected from three sources: the Dan-Petach Tikva district Clalit database, the RMC database and medical records, and the Tel Aviv Sourasky Medical Center database and medical records.

Assessment of BRAF V600E mutation status was performed by using a deoxyribonucleic acid (DNA) isolation kit and targeted next-generation sequencing (NGS), as described below.

### Outcomes

The primary endpoint of this study was an investigator-assessed ORR based on positron emission tomo-graphy (PET)/computerized tomography (CT) scans evaluated by a single radiologist prior to and during treatment. The Response Evaluation Criteria in Solid Tumors version 1.1 (RECIST v1.1) was used for this evaluation, as follows: complete response (100% disappearance of target lesion), partial response (≥30% decrease in tumor size), stable disease (<30% tumor decrease and <20% increase in tumor size), and progressive disease (≥20% increase in lesion size) (^24^).

Censoring rules to indicate ORR were as follows: patients who had an event (progressive disease) at their last date of follow-up were taken into account; if a patient died before an event occurred that date was selected, and the patient was censored. Safety, as adverse events (AEs), was assessed according to the National Cancer Institute Common Toxicity Criteria version 4.0 (CTCAE v4.0) (^25^).

### DNA isolation

Eight 5-µm-thick sections were extracted using a QIAamp DNA Mini Kit (Qiagen, Hilden, Germany) according to the manufacturer’s instructions. DNA content and quality were determined using a Qubit dsDNA assay (Invitrogen, Thermo Fisher Scientific).

### Targeted NGS

An input of 10 ng of DNA was used as a template to generate libraries using an Oncomine Solid Tumor (OST) DNA kit (Thermo Fisher Scientific, USA) following the manufacturer’s instructions. The OST panel can identify somatic mutations in 22 hotspot genes, including the BRAF gene. Sequencing was performed on an Ion GeneStudio S5 prime system (Thermo Fisher Scientific), and data were analyzed by an Ion Reporter Server system (Thermo Fisher Scientific).

### Statistical analysis

Continuous variables with nonnormal distributions are presented as medians and interquartile ranges (IQRs). Dichotomous or nominal categorical variables are presented as absolute numbers and percentages. Overall survival (calculated from the date of diagnosis) and disease-free survival analyses were conducted using Kaplan-Meier analysis. Statistical significance was defined as a p value ≤ 0.05, and all the statistical analyses were performed using SPSS version 23 (IBM).

## RESULTS

### Baseline characteristics

Baseline demographics and clinical characteristics are presented in **Table 1**. The median patient age was 68 years at the initial diagnosis, and 60% of the patients were men. Overall, 48.3% of patients had clinically relevant baseline comorbidities, with hypertension being the most common disease in 60% of patients, followed by dyslipidemia in 50% of patients, ischemic heart disease and diabetes in 40% of patients, and chronic kidney disease in 20% of patients (**Table 1**). The median duration of treatment was 14 months.

**Table 1:** Baseline demographic and clinical characteristics

Characteristic	Patients (N = 10)
Age at initial diagnosis, years (median, IQR)	68 (58.5-73.5)
Age at diagnosis of incurable disease, years (median, IQR)	68 (59-73)
Men, n (%)	6 (60%)
**Comorbidities**	**29 (48.3%)**
Hypertension, n (%)	6 (60%)
Ischemic heart disease, n (%)	4 (40%)
Chronic kidney disease, n (%)	2 (20%)
Dyslipidemia, n (%)	5 (50%)
Diabetes mellitus, n (%)	4 (40%)

### Disease and treatment characteristics

All patients were diagnosed with PTC and treated with RAI at a median dosage of 300 mCi (**Table 2**). All patients had lung metastases, with mediastinal metastases in 50% of patients, bone metastases in 30% of patients, and liver metastases in 10% of patients. Two patients received SBRT to the lungs, and four patients received EBRT to mediastinum, neck and bone metastases.

**Table 2:** Disease and treatment characteristics

	Patients (N = 10)
**Metastasis Location**	
Neck, n (%)	6 (60%)
Lungs, n (%)	10 (100%)
Mediastinum, n (%)	5 (50%)
Bone, n (%)	3 (30%)
Liver, n (%)	1 (10%)
Brain, n (%)	0
**Cumulative RAI dose, mCi (N = 10), median (IQR)**	300 (175-385)
**External beam radiotherapy, n (%)**	7 (70%)
**BRAF inhibitor alone, n (%)**	1 (10%)
**BRAF plus MEK inhibitors, n (%)**	9 (90%)
**Line of Treatment**	
First-line, n (%)	3 (30%)
Second-line, n (%)	6 (60%)
Third-line, n (%)	1 (10%)

One patient (10%) received a BRAF inhibitor alone, and 90% of patients received the combination of BRAF and MEK inhibitors (**Table 2**). Thirty percent of patients received combination therapy with dabrafenib plus trametinib as first-line therapy and 60% of patients received either dabrafenib alone or dabrafenib plus trametinib (10% and 50% of patients, respectively) as second-line therapy. Ten percent of patients received dabrafenib plus trametinib as third-line therapy (**Table 2**).

The mean time to first-line therapy (defined as the time from diagnosis to the date of first line therapy after diagnosis as an incurable disease) was 70 ± 53.31 months, the mean time from firstto second-line therapy (70% of patients) was 20 ± 9 months, and the mean time from secondto third-line therapy (20% of patients) was 29 ± 31.11 months (data not shown).

Patients who did not receive dabrafenib or dabrafenib plus trametinib as first-line therapy received either lenvatinib (50% of patients) or sorafenib (20% of patients). Ten percent of patients discontinued therapy after 1 month because of side effects; however, this patient was still included because he was part of the intent-to-treat (ITT) overall population.

### Treatment outcome

A total of 90% of patients were evaluable, as the imaging results of 1 patient could not be classified because this patient discontinued therapy after 1 month due to side effects (**Table 3**). After treatment, 20% of patients had a complete response, 50% of patients had a partial response, 1 patient had stable disease, and 1 patient had progressive disease (**Table 3**). A total of 70% of patients had an ORR (complete or partial response). The median duration of response was 10.5 months. During continued follow-up from the start of dabrafenib and trametinib treatment until the end of follow-up (regardless of temporary interruption or treatment cessation), 70% of patients died.

**Table 3:** Treatment outcomes

	Patients (N = 10)
**Best Response**	
Complete response, n (%)	2 (20%)
Partial response, n (%)	5 (50%)
Stable disease, n (%)	1 (10%)
Progressive disease, n (%)	1 (10%)
n/e, n (%)	1 (7.7%)
**Treatment Duration, median (IQR)**	10.5 (4.75-29.5)

**Figure 1:**
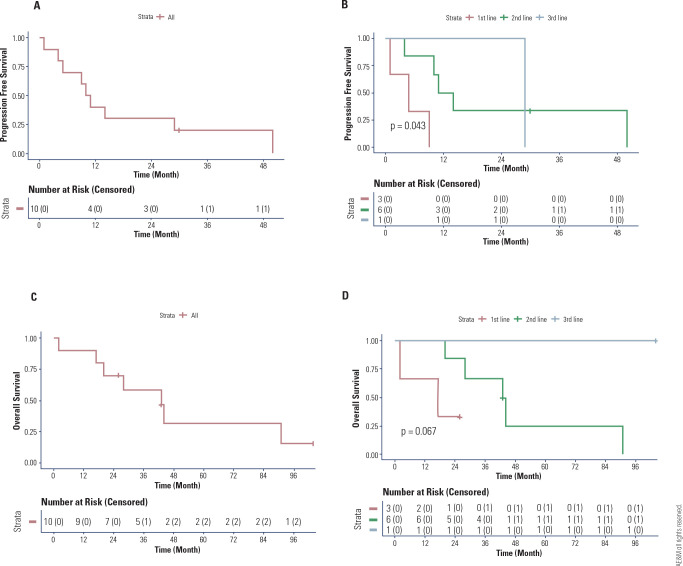
Kaplan-Meier curves of progression-free survival (PFS) and overall survival (OS). PFS in (**A**) all patients and (**B**) those grouped by therapeutic group. OS in (**C**) all patients and **(D**) those grouped by therapeutic group.

The 24-month PFS rate was greater than 25% (**Figure 1A**), and the 24-month OS rate was less than 75% (**Figure 1C**). PFS was 70%, 40%, 30%, and 30% at 6, 12, 18, and 24 months, respectively (**Figure 1**A). In the first-line therapy group, 3 patients received dabrafenib plus trametinib, and the PFS was 33.3% at 6 months and 0% at 12, 18, and 24 months (**Figure 1B**). In the second-line therapy group, for 6 patients who received either dabrafenib (1 patient) or dabrafenib plus trametinib (5 patients), the PFS rates were 83.3%, 50%, 33.3%, and 33.3% at 6, 12, 18, and 24 months, respectively (**Figure 1B**). In the third-line therapy group, with 1 patient receiving dabrafenib plus trametinib, the PFS was 100% at 6, 12, 18, and 24 months and 0% at 48 months (**Figure 1B**). A significant difference in PFS was observed among the 3 different lines of therapy (p = 0.043) (**Figure 1B**).

The overall survival rates were 90%, 90%, 80%, and 70% at 6, 12, 18, and 24 months, respectively (**Figure 1C**). In the first-line therapy group, the OS was 66.6% at 6 and 12 months and 33.3% at 18 and 24 months (**Figure 1D**). In the second-line therapy group, the OS was 100% at 6, 12, and 18 months and 83.33% at 24 months (**Figure 1D**). In the third-line therapy group, the OS was 100% until 96 months (**Figure 1D**). A significant difference in OS was observed among the 3 different lines of therapy (p = 0.024) (**Figure 1D**).

One patient experienced symptomatic pulmonary miliary dissemination and was treated with lenvatinib with a partial response; progression under lenvatinib prompted a switch in treatment to dabrafenib and trametinib. However, the regimen was discontinued after only 42 days because of adverse effects, including persistent fever and chills that were unresponsive to conservative management. Consequently, the patient elected to discontinue the treatment.

Treatment-related adverse events were common and led to drug discontinuation in 2 patients (20%), treatment interruption in 4 patients (40%), and dose reduction in 4 patients (40%). The most frequently reported AEs included pyrexia (70%), fatigue (50%), rash (30%), elevated liver enzymes (20%), stomatitis (10%), and acute kidney injury (10%). Grade ≥ 3 AEs were observed in 4 patients (40%).

## DISCUSSION

This study reports clinical efficacy results of 10 patients diagnosed with progressive, metastatic BRAF V600E-mutated RAI-refractory thyroid cancer treated with either dabrafenib (a BRAF inhibitor) alone or a combination of dabrafenib and trametinib (BRAF and MEK inhibitors) as first-, secondor third-line therapy. The key results indicate an ORR of 70%, with a median treatment duration of 10.5 months.

Previous studies have indicated that the 10-year OS in patients diagnosed with metastatic RAI-refractory thyroid cancer is as low as 10% in patients with no RAI uptake and 29% in patients who have RAI uptake but who develop RAI-resistant disease (^5^), and a median survival of only 3-5 years is observed in these patients after the diagnosis of metastatic disease (^26^). In patients with metastatic BRAF-mutated disease, use of dabrafenib monotherapy as first-line therapy resulted in a median progression-free survival of 11.3 months (^27^). Moreover, only a few studies have evaluated the outcomes of patients with metastatic BRAF-mutated disease previously treated with multikinase inhibitors. A study by Brose and cols. demonstrated only 27.3% partial response with vemurafenib (a BRAF inhibitor), with 55% mortality within one year of treatment, and a median progression-free survival of 8.9 months (^28^). Compared with these studies, our study demonstrated improved response rates and progression free survival when dabrafenib and trametinib were used, especially in patients previously treated with multikinase inhibitors. Until recently, lenvatinib, a multityrosine kinase inhibitor, was recommended as the first-line therapy for most patients diagnosed with RAI-refractory thyroid cancer (^18^,^29^). However, currently, the European Society for Medical Oncology (ESMO) and the National Comprehensive Cancer Network (NCCN) recognize the importance of somatic genome analysis, including BRAF V600E mutation screening, in patients diagnosed with RAI-refractory thyroid cancer and recommend combination therapy with dabrafenib and trametinib in patients harboring this mutation (^30^). The role of BRAF/MEK inhibitors in resensitizing RAI-refractory thyroid cancer to iodine has also been promising, as selumetinib (a MEK inhibitor) (^31^) and dabrafenib (^32^) have been shown to increase iodine uptake and retention in patients diagnosed with RAI-refractory thyroid cancer. This inability of cancer cells to absorb iodine results from the loss of thyroid differentiation, which is directly associated with MAPK activation and is seen more often in tumors with BRAF mutations than in tumors with other mutations (such as receptor tyrosine kinase [RTS]) (^26^), indicating the need for molecular targeted therapeutic options in RAI-refractory thyroid cancer.

The BRAF V600E mutation, which accounts for more than 90% of all BRAF mutations, is an enzyme (a serine-threonine kinase) that activates MEK1 and MEK2 (^33^) and is expressed abundantly in the follicular cells of the thyroid (^34^). Thus, a BRAF V600E mutation induces dysregulation of the MAPK pathway, which is responsible for cell proliferation and differentiation, in thyroid follicular cells, ultimately producing an aggressive PTC with a very poor prognosis. The mechanism underlying the role of dabrafenib in treating PTC has been associated with targeting the proteins (RAF kinases) produced by the BRAF V600E mutation, which results in MEK phosphorylation, cell cycle arrest and cancer cell apoptosis (^35^). However, numerous studies have indicated that resistance to dabrafenib monotherapy can develop via secondary genetic alterations and expression, increasing MAPK signaling and continued cancer growth (^36^). Another observation of dabrafenib monotherapy was that a large number of patients were diagnosed with secondary skin cancers, which were associated with contradictory activation of the MAPK pathway in nonmutant BRAF cells (^37^). These findings led to the therapeutic option of the combination of dabrafenib plus trametinib in an attempt to block the MAPK pathway at 2 different points - dabrafenib used as a BRAF inhibitor and trametinib as a MEK1/2 inhibitor, inducing oncogenic cell cycle arrest (^38^). The use of dabrafenib in combination with trametinib has also been emphasized in other BRAF V600E-mutated cancers, such as stage III melanomas, where this combined therapy was shown to significantly improve the OS rate and disease progression in comparison with patients receiving the standard of care (^39^). Despite these data, a recent phase II study published by Busaidy and cols. (^17^) compared treatment with dabrafenib alone or dabrafenib plus trametinib in patients with BRAF-mutated DTC and found that the combination was not superior in efficacy to dabrafenib monotherapy. Of note, the study was probably underpowered (53 patients), as the combination group had a PFS of 15.1 months compared with 10.7 months with dabrafenib monotherapy, which was not statistically significant.

In the present study, the combined therapy with dabrafenib and trametinib was well tolerated, with general weakness, pyrexia, and eczematous rash as the most common AEs, which is in accordance with previous studies in which pyrexia, anemia, decreased appetite, fatigue, and nausea were observed (^14^,^15^). Amaria and cols. also reported no serious treatment-related AEs, with grade 1 to 2 toxicities in patients diagnosed with stage III melanomas who received dabrafenib in combination with trametinib over a mean period of 18.6 months (^39^).

Although the present study is limited by its small sample size, these results are still important when focusing on the current need for effective secondand third-line therapeutic options in targeting this aggressive cancer.

In conclusion, the use of dabrafenib in combination with trametinib resulted in a substantial clinical benefit, with notable PFS and sustained OS in patients diagnosed with metastatic BRAF V600E-mutated, RAI-refractory PTC. These findings support the rationale to further investigate targeted combination therapeutic options for this aggressive disease.

## Data Availability

datasets related to this article will be available upon request to the corresponding author.
